# Glass Formation, Chemical Properties and Surface Analysis of Cu-Based Bulk Metallic Glasses

**DOI:** 10.3390/ijms12042275

**Published:** 2011-04-04

**Authors:** Chunling Qin, Weimin Zhao, Akihisa Inoue

**Affiliations:** 1 School of Materials Science and Engineering, Hebei University of Technology, Tianjin 300132, China; E-Mail: dashan_qin@yahoo.com; 2 WPI Advanced Institute for Materials Research, Tohoku University, Sendai 980-8577, Japan; E-Mail: ainoue@imr.tohoku.ac.jp

**Keywords:** metallic glasses, glass formation, chemical properties, polarization curves, XPS measurements

## Abstract

This paper reviews the influence of alloying elements Mo, Nb, Ta and Ni on glass formation and corrosion resistance of Cu-based bulk metallic glasses (BMGs). In order to obtain basic knowledge for application to the industry, corrosion resistance of the Cu–Hf–Ti–(Mo, Nb, Ta, Ni) and Cu–Zr–Ag–Al–(Nb) bulk glassy alloy systems in various solutions are reported in this work. Moreover, X-ray photoelectron spectroscopy (XPS) analysis is performed to clarify the surface-related chemical characteristics of the alloy before and after immersion in the solutions; this has lead to a better understanding of the correlation between the surface composition and the corrosion resistance.

## 1. Introduction

Bulk metallic glasses (BMGs) have been drawing increasing attention in recent years due to their superior properties and potential as new engineering materials [1,2]. To date, a large number of BMG systems, including Mg-, Zr-, Pd-, Fe-, Co-, Ti-, Ni-, and Cu-based multicomponent systems, have been produced by conventional mold casting and water quenching techniques [3–10]. Among them, the Cu-based BMGs are particularly interesting and show commercial potential as structural materials in some fields due to the combination of high strength, distinct plasticity and relatively low cost of fabrication. This includes the discoveries of unusual glass-forming ability (GFA) and high strength in ternary Cu–Zr(Hf)–Ti(Al) [10–12] and quaternary Cu–Zr–(Ti, Al)–Ag [13–15] alloys. These BMGs—as an engineering material—are now attracting our attention on applications in the industrial field. However, so far, extensive studies have focused on the glass formation, crystallization processes, mechanical, and physical properties of BMGs. There is quite some corrosion data of BMGs in aqueous solutions. Actually, chemical properties of materials are of great importance. In order to enable the use of the new type of BMGs as engineering material, the glassy alloys must have a good corrosion resistance and electrochemical stability in industrial environments. Therefore, it is of great importance to investigate the corrosion behavior and, subsequently, to improve the corrosion resistance by alloying corrosion resistant elements, such as Mo, Nb and Ta, *etc*. Moreover, a better understanding of the role of the alloy constituent elements in corrosion resistance is useful for designing a high corrosion resistant BMG alloy.

This paper aims to review the influence of alloying elements Mo, Nb, Ta and Ni on glass formation and corrosion resistance of Cu-based BMGs. In order to obtain basic data for application to the industry, corrosion resistance of the Cu–Hf–Ti–(Mo, Nb, Ta, Ni) and Cu–Zr–Ag–Al–(Nb) glassy alloy systems in various solutions are reported in this paper. Moreover, X-ray photoelectron spectroscopy (XPS) is used to clarify the origin of the high corrosion resistance of the alloys. Particular attention has been paid to the chemical composition and the oxidation states of alloy constituents in surface films.

## 2. Results and Discussion

### 2.1. Fabrication, Glass Formation and Corrosion Resistance of the Cu–Hf–Ti–(Mo, Nb, Ta) BMGs

The Cu-based BMGs are known for their ultrahigh strength exceeding 2 GPa, high glass-forming ability (GFA), and good wear resistance, allowing potential applications as advanced engineering materials in many areas, such as surgical instruments and bipolar plates in fuel cells. However, the corrosion resistance of these Cu-based BMGs is not always better than that of conventional Cu-based crystalline alloys, though the tensile strength levels are 2- to 5-times higher for the Cu-based BMGs [16]. Therefore, there is a need to improve their corrosion resistance by alloying corrosion resistant elements to make the Cu-based BMGs acceptable for use on an industrial level.

#### 2.1.1. Effects of Additional Elements Mo, Nb and Ta on the Glass Formation and Corrosion Resistance

**Figure 1.**
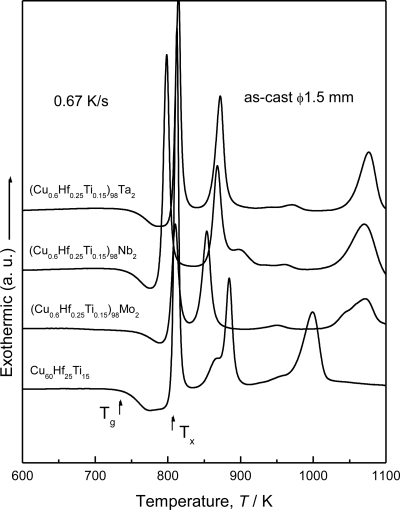
DSC curves of the as-cast (Cu_0.6_Hf_0.25_Ti_0.15_)_98_M_2_ (M = Mo, Nb and Ta) glassy alloys.

Firstly, we examined the effect of additional elements Mo, Nb and Ta on glass formation of the Cu–Hf–Ti–(Mo, Nb and Ta) alloys. Figure 1 shows the differential scanning calorimetry (DSC) curves of the Cu-based bulk glassy alloys containing Mo, Nb and Ta elements with a diameter of 1.5 mm, together with the data of the Cu_60_Hf_25_Ti_15_ alloy [17]. All the alloys exhibit the distinct glass transition, followed by a large supercooled liquid region before crystallization. The temperature interval of the supercooled liquid region (Δ*T*_x_) of the Cu_60_Hf_25_Ti_15_ glassy alloy is 60 K. By the addition of a small amount of Mo, Nb or Ta, the Δ*T*_x_ decreases, but still keeps large values of 51 K at 2 at.% Ta, 46 K at 2 at.% Nb and 40 K at 2 at.% Mo. The critical diameter for glass formation is 4.0 mm at 2 at.% Nb, 3.5 mm at 2 at.% Ta and 1.5 mm at 2 at.% Mo.

The average corrosion rates of the Cu–Hf–Ti–(Mo, Nb, Ta) bulk glassy alloys with a diameter of 1.5 mm in 1 N HCl, 3 mass% NaCl and 1 N H_2_SO_4_ + 0.001 N NaCl solutions at 298 K for 168 h are shown in Table 1 [18]. The accuracy of the balance for measuring weight loss of all the samples in this paper is about 1 × 10^−5^ g. In 1 N HCl solution, the corrosion rate of the Cu_60_Hf_25_Ti_15_ alloy is about 0.340 mm year^−1^ and the decrease in corrosion rates of the alloys by substituting 2 at.% Mo, Nb or Ta is about half of that of the Cu_60_Hf_25_Ti_15_ alloy. In 3 mass% NaCl solution, the corrosion rates of the Cu–Hf–Ti–(Mo, Nb, Ta) alloys decrease significantly from the order of 10^−1^ mm year^−1^ for the Cu_60_Hf_25_Ti_15_ alloy to the order of 10^−3^ mm year^−1^ for the Cu–Hf–Ti–(Mo, Nb, Ta) alloys. In 1 N H_2_SO_4_ + 0.001 N NaCl, the alloys containing additional elements show undetectable weight loss, indicating a corrosion rate of less than 1 × 10^−3^ mm·year^−1^, which is the reproducibility limit for the present measurement.

**Figure 2.**
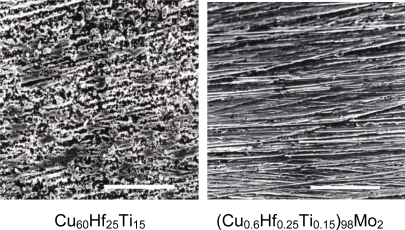
SEM micrographs of the surfaces of the bulk glassy (Cu_0.6_Hf_0.25_Ti_0.15_)_100−x_Mo_x_ (x = 0 and 2 at%) alloys after immersion in 3 mass% NaCl solution for 168 h at 298 K.

After immersion in 3 mass% NaCl solution for one week, changes in surface morphology of the samples were further observed by SEM, as shown in Figure 2 [18]. The alloy without any additional elements suffered serious corrosion, and its surface was covered with thick corrosive products. The 2 at% Mo, Nb and Ta alloy still kept the previous metallic luster and almost no changes in their surfaces were seen after immersion. This indicates that the addition of Mo, Nb or Ta to the Cu–Hf–Ti alloy is effective for suppressing the corrosion of the Cu-based glassy alloys in the chloride containing environments.

#### 2.1.2. Fabrication, GFA and Corrosion Resistance of Cu–Hf–Ti–Nb BMGs

In Section 2.1.1, we demonstrated that the addition of 2 at.% Mo, Nb or Ta to the Cu–Hf–Ti bulk glassy alloy was effective in improving the corrosion resistance of the alloys. However, the corrosion resistance of these glassy alloys is still low in acidic and chloride containing solutions. Moreover, the further addition of Mo or Ta to the alloys results in a significant decrease in the glass-forming ability (GFA). Therefore, it is expected that Cu–Hf–Ti–Nb glassy alloys synthesized by rapid solidification can show high GFA and improved corrosion resistance.

##### 2.1.2.1. Fabrication and Thermal Properties of Cu–Hf–Ti–Nb BMGs

**Figure 3.**
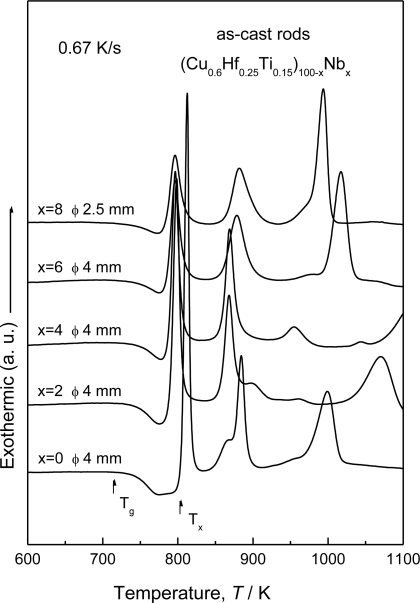
DSC curves of the as-cast (Cu_0.6_Hf_0.25_Ti_0.15_)_100−x_Nb_x_ (x = 0, 2, 4, 6 and 8 at.%) glassy alloys with their critical diameters for glass formation.

**Figure 4.**
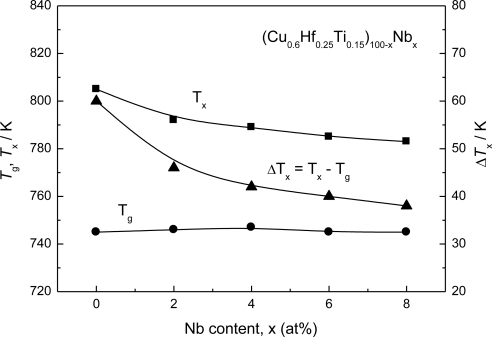
Changes in *T*_g_, *T*_x_ and Δ*T*_x_ as a function of Nb content for the as-cast (Cu_0.6_Hf_0.25_Ti_0.15_)_100−x_Nb_x_ glassy alloys.

Figure 3 shows DSC curves of the (Cu_0.6_Hf_0.25_Ti_0.15_)_100−x_Nb_x_ bulk glassy alloys with their critical diameters for glass formation, where *T*_g_ and *T*_x_ correspond to glass transition temperature and onset temperature of crystallization, respectively [19]. The distinct glass transition, followed by a supercooled liquid region prior to crystallization, is recognized. The crystallization of the Cu–Hf–Ti–Nb alloys with a supercooled liquid region appears to occur through multiple exothermic events, indicating a multi-stage phase transformation process. The *T*_g_, *T*_x_ and Δ*T*_x_ are plotted against Nb content in the alloys in Figure 4 [19]. As the Nb content increases, the glass transition temperature (*T_g_*) remains almost constant while crystallization temperature (*T*_x_) decreases, resulting in a decrease in Δ*T*_x_ (= *T*_x_ − *T*_g_) from 60 K at 0 at.% Nb to 38 K at 8 at.% Nb. Although the addition of Nb to the alloys exhibits a negative effect on Δ*T*_x_, the Cu–Hf–Ti–Nb bulk glassy alloys still keep high GFA. Actually, the critical diameter for glass formation (*d*_c_) was 4 mm for the alloys with Nb contents up to 6 at.%, and 2.5 mm for the 8 at.% Nb alloy. The outer surfaces of the cast rods were smooth and no trace of precipitation of crystalline phase was seen.

##### 2.1.2.2. Corrosion Behavior of Cu–Hf–Ti–Nb Bulk Glassy Alloys

**Figure 5.**
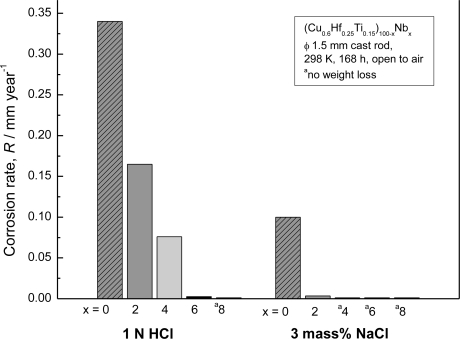
The average corrosion rates of the as-cast (Cu_0.6_Hf_0.25_Ti_0.15_)_100−x_Nb_x_ glassy alloys (x = 0, 2, 4, 6 and 8 at.%) in 1 N HCl and 3 mass% NaCl solutions at 298 K open to air.

In Figure 5 [19], the average corrosion rates of the as-cast (Cu_0.6_Hf_0.25_Ti_0.15_)_100−x_Nb_x_ glassy alloys immersed in 1 N HCl and 3 mass% NaCl solutions at 298 K open to air for one week are plotted as a function of Nb content. In 1 N HCl solution, it is clearly seen that the alloy without Nb dissolves actively, showing a high corrosion rate of 0.34 mm·y^−1^. The alloys containing Nb exhibit lower corrosion rates of 0.17 mm·y^−1^ at 2 at.% Nb, 7.6 × 10^−2^ mm·y^−1^ at 4 at.% Nb, 2.6 × 10^−3^ mm·y^−1^ at 6 at.% Nb, and less than the detectable value at 8 at.% Nb. This means that the average corrosion rate of 8 at.% Nb alloy is less than 1 × 10^−3^ mm·y^−1^, which was the reproducibility limit for the present measurement. On the other hand, in 3 mass% NaCl solution, it also appears that the average corrosion rate of the Cu–Hf–Ti–Nb glassy alloys decreases sharply with the addition of the alloying element Nb and becomes less than 1 × 10^−3^ mm·y^−1^ when the Nb content reaches 4 at.% or higher.

**Figure 6.**
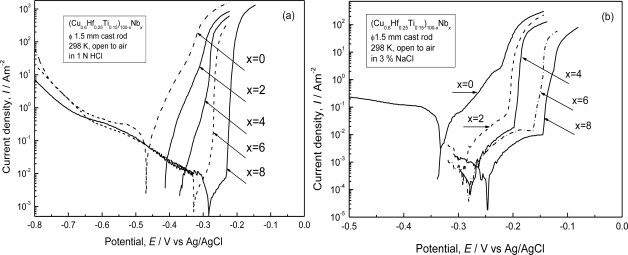
Potentiodynamic polarization curves of the as-cast (Cu_0.6_Hf_0.25_Ti_0.15_)_100−x_Nb_x_ glassy alloys (x = 0, 2, 4, 6 and 8 at.%) alloys in (**a**) 1 N HCl and (**b**) 3 mass% NaCl. solutions at 298 K open to air.

Figure 6 shows the polarization curves for the as-cast (Cu_0.6_Hf_0.25_Ti_0.15_)_100−x_Nb_x_ glassy alloys with a diameter of 1.5 mm in 1 N HCl and 3 mass% NaCl solutions at 298 K open to air, respectively [19]. In 1 N HCl solution in Figure 6(a), the alloys containing Nb up to 4 at.% dissolve actively due to the formation of CuCl_2_^−^ complex anion [20]. However, the alloys containing 6 at.% Nb and more show distinct spontaneous passivation although they suffer pitting by anodic polarization. All the alloys exhibit similar cathodic polarization behavior. On the other hand, the addition of Nb to the alloys ennobles the open circuit potential and enhances the corrosion resistance in the 1 N HCl solution. In 3 mass% NaCl solution, as shown in Figure 6(b), the glassy alloys containing Nb are spontaneously passivated and their passive current densities are in the range of 10^−2^–10^−3^ A·m^−2^ before the occurrence of pitting corrosion, which is indicated by the abrupt rise in the current density. It is noticed that the alloys with a larger amount of Nb show the nobler pitting potential, leading to the reduction of pitting susceptibility and improvement of pitting corrosion resistance. In addition, the addition of Nb positively affects the passivating ability of the alloys by decreasing their passive current densities and widening their passive regions. On the other hand, all the Cu–Hf–Ti–Nb glassy alloys with Nb content up to 8 at.% have undetectable weight loss with the present measurement after immersion in 1 N H_2_SO_4_ and 1 N H_2_SO_4_ + 0.001 N NaCl solutions for two weeks, revealing that the Nb-containing Cu–Hf–Ti–Nb alloys possess high corrosion resistance in these solutions.

##### 2.1.2.3. XPS Analysis of Surface Film

For a better understanding of the effect of Nb on the corrosion resistance of the (Cu_0.6_Hf_0.25_Ti_0.15_)_100−x_Nb_x_ glassy alloys, X-ray photoelectron spectroscopy analysis (XPS) was performed for the specimens exposed to air after mechanical polishing and those immersed in 1 N HCl and 3 mass% NaCl solutions open to air for one week. Over a wide binding energy region, the XPS spectra exhibited peaks of copper, titanium, hafnium, niobium, oxygen and carbon. The C 1s peaks were those from so-called contaminant carbon covering the top surface of the specimen. The O 1s spectrum consisted of peaks originating from oxygen in metal-O-metal bond, metal-OH bond and bound water. The peaks of Cu 2p, Hf 4f, Ti 2p, Nb 3d were composed of peaks corresponding to their oxidized states in a surface film and the metallic states in an underlying alloy surface. The major cations in the surface film were Cu^+^, Cu^2+^, Hf^4+^, Ti^4+^ and Nb^5+^.

**Figure 7.**
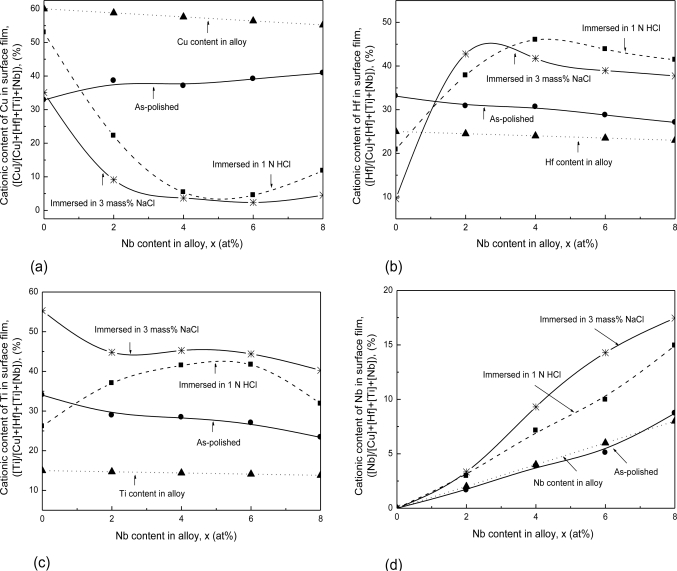
Cationic content of (**a**) Cu; (**b**) Hf; (**c**) Ti; and (**d**) Nb, in surface film as a function of Nb content for the (Cu_0.6_Hf_0.25_Ti_0.15_)_100−x_Nb_x_ bulk glassy alloys exposed to air and those immersed in 1 N HCl and 3 mass% NaCl solutions open to air for one week after mechanical polishing.

Figure 7 show the cationic contents (oxidized states) of Cu, Hf, Ti and Nb in the surface films as a function of Nb content for the (Cu_0.6_Hf_0.25_Ti_0.15_)_100−x_Nb_x_ (x = 0–8 at.%) bulk glassy alloys exposed to air and those immersed in the solutions after mechanical polishing [19]. In the surface films formed by air exposure after mechanical polishing, Hf and Ti elements are enriched, while Cu is deficient and Nb scarcely changes with respect to the alloy compositions. Accordingly, air exposure of the (Cu_0.6_Hf_0.25_Ti_0.15_)_100−x_Nb_x_ alloys results in preferential oxidation of Hf and Ti. Immersion in the NaCl and the HCl solutions causes a significant change in the compositions of the surface films for the alloys with and without Nb. The content of Cu cations in the surface film for the alloys free of Nb slightly increases after immersion in the NaCl solution and further increases after immersion in the HCl solution, while those for the Nb-containing alloys immersed in the solutions decrease significantly, as shown in Figure 7(a). Moreover, it is seen in Figure 7(b),(c) that the surface films of the Nb-free alloy immersed in the solutions are deficient in Hf cations and greatly enriched in Ti cations with respect to their alloy contents. On the other hand, both Hf and Ti cations are largely concentrated in the surface films of the Nb-containing alloys after the immersion. In Figure 7(d), it can be seen that the Nb concentration in the surface films of the alloys immersed in the HCl increases, and further increases after immersion in the NaCl solution, in comparison with that in the air-formed films or the alloy Nb content. Furthermore, the Nb concentration in the surface films of the alloys immersed in the solutions significantly increases with an increase in Nb content in the alloys. Thus, Hf, Ti and Nb cations are largely concentrated in the surface for the alloys immersed in the above solutions. The enrichment of Hf, Nb and Ti might be explained by preferential dissolution of Cu and the effect of Nb-addition. As indicated by the results of the corrosion rates and electrochemical properties of the present alloys in Figures 5 and 6, we can see the alloy with a higher Nb content exhibits the higher passivating ability, and hence better corrosion resistance. The protective quality of the surface films is improved by increasing the Nb content. This is in agreement with XPS surface analysis. Accordingly, the formation of the highly protective Hf-, Ti- and Nb-enriched surface film on the alloys by immersion in HCl and NaCl solutions could be responsible for the high corrosion resistance of the Nb-containing alloys.

### 2.2. Fabrication, Thermal Properties and Corrosion Resistance of Cu–Hf–Ti–Ni–Nb BMGs

In our previous work shown in Sections 2.1.1 and 2.1.2, the addition of Mo, Nb or Ta is shown to effectively improve the corrosion resistance of the Cu–Hf–Ti-based BMGs in various aggressive environments. Unfortunately, the supercooled liquid region (Δ*T*_x_) decreases greatly with alloying additional elements, resulting in a decrease in GFA and thermal stability of the supercooled liquid before crystallization. Hence, the limitations of thermal stability and composition range for the glass formation for the Cu–Hf–Ti-based alloys will prevent a further extension of their application fields. Great efforts have been devoted to develop new BMGs with larger supercooled liquid region and good viscous flow workability in conjunction with higher corrosion resistance. Recently, we found that the addition of 5 at.% Ni to Cu–Hf–Ti-based BMGs caused a significant increase in the supercooled liquid region and the extension of the composition range for the bulk metallic glass formation. In addition, the simultaneous addition of Ni and Nb elements is effective for the improvement of corrosion resistance in the maintenance of high GFA.

#### 2.2.1. The Effects of the Ni and Nb Additions on GFA and Thermal Stability

**Figure 8.**
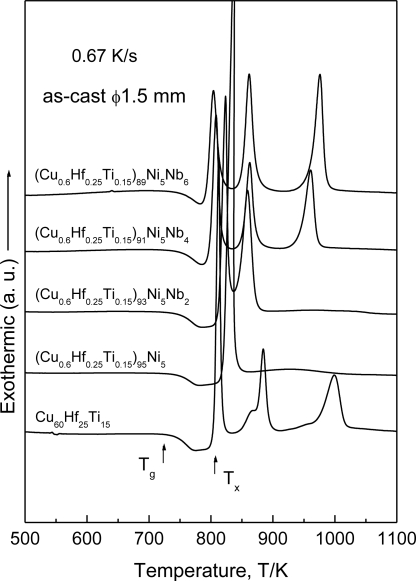
DSC curves of the as-cast (Cu_0.6_Hf_0.25_Ti_0.15_)_100−x−y_Ni_y_Nb_x_ (x = 0 to 6 at.% and y = 0 to 5 at.%) glassy rods with a diameter of 1.5 mm.

XRD patterns identified that bulk alloys with a diameter of 1.5 mm, consisting of a glassy single phase without crystallinity, were fabricated in a wide composition range of 0 to 5 at.% Ni and 0 to 6 at.% Nb for the (Cu_0.6_Hf_0.25_Ti_0.15_)_100−x−y_Ni_y_Nb_x_ alloys. Figure 8 shows DSC curves of the (Cu_0.6_Hf_0.25_Ti_0.15_)_100−x−y_Ni_y_Nb_x_ BMG rods with a diameter of 1.5 mm, where *T*_g_ and *T*_x_ correspond to glass transition temperature and onset temperature of crystallization, respectively [21]. The addition of the Ni element causes an extension of a supercooled liquid region (Δ*T*_x_ = *T*_x_ − *T*_g_) from 60 K for Cu_60_Hf_25_Ti_15_ to 70 K for (Cu_0.6_Hf_0.25_Ti_0.15_)_95_Ni_5_, accompanying the change in the crystallization mode from multiple stages to a single stage. It can be concluded that the addition of 5 at.% Ni is beneficial for increasing the thermal stability of the supercooled liquid before crystallization. A larger supercooled liquid region and a higher stability of the supercooled liquid phase are useful for the superplastic processing of the alloy in supercooled liquid state to produce, for example, bipolar plates for fuel cells or intricate devices. With further alloying Nb element to the (Cu_0.6_Hf_0.25_Ti_0.15_)_95_Ni_5_ alloy, the glass transition temperature (*T_g_*) remains almost constant while crystallization temperature (*T*_x_) decreases, resulting in a decrease in Δ*T*_x_ (=*T*_x_ − *T*_g_) from 70 K at 0 at.% Nb to 40 K at 6 at.% Nb. Moreover, the distinguished single-stage exothermic reaction due to crystallization changes to multiple exothermic reactions. The critical diameter for glass formation (*d_c_*) was 2 mm for 5 at.% Ni alloy, though the alloy exhibits the largest Δ*T*_x_ value of 70 K, 3 mm for the alloys with Nb content up to 4 at.%, and 2 mm for 6 at.% Nb alloy. It is interesting to note that the addition of Nb to the (Cu_0.6_Hf_0.25_Ti_0.15_)_95_Ni_5_ quaternary alloy promotes the glass-forming ability, despite the fact that the Nb element negatively influences the supercooled liquid region of the Cu–Hf–Ti–Ni–Nb BMGs.

#### 2.2.2. The Effects of the Ni and Nb Additions on Corrosion Resistance

**Figure 9.**
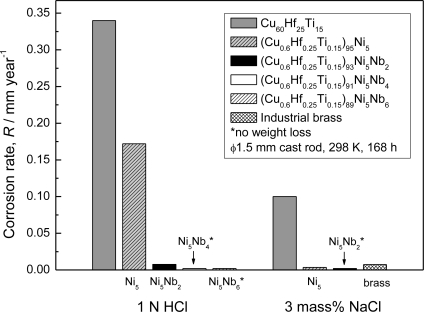
Corrosion rates of the Cu–Hf–Ti–Ni–Nb BMGs in 1 N HCl and 3 mass% NaCl solutions at 298 K open to air.

Figure 9 shows the average corrosion rates of the as-cast (Cu_0.6_Hf_0.25_Ti_0.15_)_100−x−y_Ni_y_Nb_x_ alloys with a diameter of 1.5 mm immersed in 1 N HCl and 3 mass% NaCl solutions at 298 K open to air for one week, respectively [21]. The corrosion rate of an industrial brass (60 wt.% Cu + 40 wt.% Zn) in 3 mass% NaCl is also displayed for comparison. The corrosion rate of less than 1 × 10^−3^ mm·year^−1^ is out of the detectable limits for the present measurements and hence is marked with a star in Figure 9.

In 1 N HCl solution, the addition of Ni or Nb causes a significant decrease in corrosion rate from 0.34 mm·year^−1^ for Cu_60_Hf_25_Ti_15_ to 0.17 mm·year^−1^ for (Cu_0.6_Hf_0.25_Ti_0.15_)_95_Ni_5_ and 0.0075 mm·year^−1^ for (Cu_0.6_Hf_0.25_Ti_0.15_)_93_Ni_5_Nb_2_. The corrosion rates are less than 1 × 10^−3^ mm·y^−1^ when the alloys reach critical composition, that is, (Cu_0.6_Hf_0.25_Ti_0.15_)_91_Ni_5_Nb_4_ alloy in 1 N HCl solutions. The effect of additional elements on the Cu–Hf–Ti alloy is more pronounced in 3 mass% NaCl solution. By substitution of 5 at.% Ni for the Cu_60_Hf_25_Ti_15_ alloy, the corrosion rate of alloy decreases by two orders of magnitude as compared to that of the Ni-free alloy in 3 mass% NaCl solution. Moreover, all the alloys show undetectable weight loss with the subsequent addition of 2 at.% Nb or more to the (Cu_0.6_Hf_0.25_Ti_0.15_)_95_Ni_5_ in this solution. It is well-known that industrial brass exhibits high corrosion resistance in sea water. The corrosion resistance of the 5 at.% Ni alloy is comparable to that of industrial brass in 3 mass% NaCl, and the corrosion resistance of the alloys by the simultaneous addition of Ni and Nb elements is better than that of the industrial brass. On the other hand, in 1 N H_2_SO_4_ + 0.01 N NaCl solution (not shown in Figure 9), a simulated fuel cell environment, no appreciable loss in sample weight is obtained for the (Cu_0.6_Hf_0.25_Ti_0.15_)_100−x−y_Ni_y_Nb_x_ (x = 0 to 6 at.% and y = 5 at.%) alloys, indicating that the present alloys with Ni or Nb possess high corrosion resistance in this solution, while the corrosion rate of the alloy without Ni or Nb is about 0.045 mm·year^−1^.

**Figure 10.**
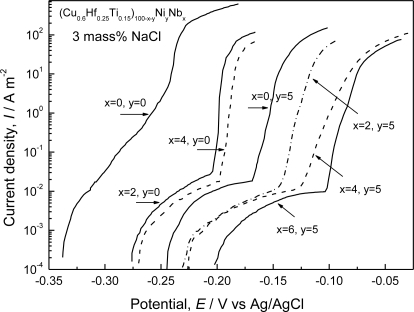
Anodic polarization curves of the Cu–Hf–Ti–Ni–Nb BMGs in 3 mass% NaCl solution at 298 K.

Further examination was conducted by potentiodynamic polarization measurements. Figure 10 shows anodic polarization curves for the as-cast 1.5 mm (Cu_0.6_Hf_0.25_Ti_0.15_)_100−x−y_Ni_y_Nb_x_ glassy alloys in 3 mass% NaCl solution at 298 K [21]. It is observed that the alloys with and without Nb or Ni show different polarization curves. The alloys containing additional elements Nb or Ni are spontaneously passivated with low anodic passive current density, although they suffer pitting corrosion by anodic polarization. In particular, the simultaneous addition of Ni and Nb to the alloys is effective in enhancing the pitting corrosion resistance in chloride-ions-containing solutions. Their pitting corrosion potentials are nobler with an increase in Nb content. Accordingly, the coexistence of Ni and Nb elements leads to the reduction of pitting susceptibility and improvement of pitting corrosion resistance of the Cu–Hf–Ti–Ni–Nb alloys. Actually, (Cu_0.6_Hf_0.25_Ti_0.15_)_89_Ni_5_Nb_6_ alloy has the highest pitting corrosion potential in comparison with the other Cu-based BMGs, such as Cu–Zr(Hf)–Ti–(Mo, Nb, Ta) and Cu–Zr–Ti–Ni–Nb alloys [17,19,22,23]. As a result, the Cu–Hf–Ti–Ni–Nb BMGs with the coexistence of Ni and Nb possess significantly lower corrosion rates in 1 N HCl, 3 mass% NaCl and 1 N H_2_SO_4_ + 0.01 N NaCl solutions, and their pitting potentials in NaCl are ennobled with the further increase in Nb content, indicating that the addition of Ni and Nb is the best combination for enhancing the corrosion resistance.

#### 2.2.3. Surface Characteristics after Immersion

The chemical and engineering applications of materials generally require the synergistic effect of various elements. In this study, the corrosion resistance of Cu–Hf–Ti–Ni–Nb alloys is enhanced synergistically by coexisting alloying Ni and Nb elements in 1 N HCl, 3 mass% NaCl and 1 N H_2_SO_4_ + 0.01 N NaCl solutions as shown in Figures 9 and 10. One of the most important characteristics is ennoblement of their pitting potentials with the simultaneous addition of Ni and Nb to the Cu–Hf–Ti alloy (Figure 10). They are higher than those of (Cu_0.6_Hf_0.25_Ti_0.15_)_100−x_Nb_x_ alloys. Ni and Nb elements of the alloys have different chemical characteristics in corrosion behavior, especially in pitting corrosion. Nb is known to be an effective element in providing the strong passivating ability, whereas Ni shows high chemical reactivity in acidic solutions [24,25]. It is interesting to clarify the role of the combination of Ni and Nb elements in the passivation and corrosion mechanism of alloys in these corrosive solutions. XPS result revealed that the large enrichment of passivating elements (Hf, Ti and Nb) in the surface film for the Ni-containing alloys immersed in various solutions is caused by preferential dissolution of Ni and Cu into solutions [25,26]. The role of Cu element in the alloys in the corrosion behavior has been reported by several surface studies [19,23]. Preferential dissolution of Ni from the alloys assists the formation of passive films highly enriched in passivating elements, such as Hf, Ti and Nb. In addition, it is observed that the cationic Nb content of the surface films formed on (Cu_0.6_Hf_0.25_Ti_0.15_)_95−x_Ni_5_Nb_x_ (x = 0 to 6 at%) alloys immersed in the solutions significantly increases with increasing Nb content of the alloys, whereas the cationic Hf, Ti, and Cu contents are not so different with the change in Nb content in the alloys [21]. This fact suggests that the surface film is further modified toward more stabilized composition with a higher Nb content. Consequently, the simultaneous addition of Ni and Nb is favorable for the alloys in forming Hf-, Ti- and Nb-enriched highly protective surface films in these corrosive solutions.

### 2.3. The Electrochemical and XPS Studies of the Cu–Zr–Ag–Al–(Nb) BMGs

Very recently, a new series of (CuZr)-based BMGs with excellent glass-forming ability (GFA) and a large supercooled liquid region have been developed [14,15]. These glassy alloys possessed high fracture strength of over 1850 MPa with appreciable plasticity. The critical diameter was 10 mm for Cu_42_Zr_42_Ag_8_Al_8_, 15 mm for Cu_40_Zr_44_Ag_8_Al_8_ and Cu_38_Zr_46_Ag_8_Al_8_, and 25 mm for Cu_36_Zr_48_Ag_8_Al_8_. Compared with other BMGs discovered so far, these newly developed (CuZr)-based BMGs present unique features, which combine the ductility of Cu-based BMGs with the high GFA and the large supercooled liquid region of Zr-based BMGs. This unique combination of properties provides the new BMGs with significant potential in application fields, such as in bipolar plate materials, biomedical instruments and micro devices.

#### 2.3.1. Electrochemical Properties and Surface Characteristics of Cu–Zr–Ag–Al BMGs

The Cu_36_Zr_48_Ag_8_Al_8_ BMG with a diameter of 25 mm and high fracture strength of 1850 MPa has attracted special attention for applications. The corrosion behavior and surface characteristics of the Cu_36_Zr_48_Ag_8_Al_8_ BMG were instigated in this work.

##### 2.3.1.1. Corrosion Rates and Electrochemical Properties of the Alloys

The average corrosion rates of the as-cast 2 mm diameter Cu_36_Zr_48_Ag_8_Al_8_ BMG immersed in acidic and alkaline solutions open to air at 298 K were measured [27]. No weight loss was detected for the rod samples after immersion in 1 N H_2_SO_4_ and 1 N NaOH solutions, respectively, even after two months. This means that the corrosion rate of the alloy is less than 1 × 10^−3^ mm·y^−1^. After immersion in 1 N H_2_SO_4_ + 0.01 N NaCl solution, the corrosion rate is about 0.15 mm·y^−1^. The result reveals that the present alloy is sensitive to chloride ions, which results from the chemical instability of the Cu species and the pitting susceptibility of the Zr and Al species in the alloy. After the weight loss tests, surfaces of the alloy rods were examined by SEM. The entire alloy samples maintained metallic luster after immersion for two months in 1 N H_2_SO_4_ and 1 N NaOH, and neither change nor pitting corrosion was observed on their surfaces. However, when the rod sample was immersed in H_2_SO_4_ containing aggressive anions (chloride ions), the sample suffered corrosion attack and its surface was covered with corrosion products, appearing as granular materials. Accordingly, the new Cu_36_Zr_48_Ag_8_Al_8_ BMG possesses excellent corrosion resistance in strong acidic and alkaline solutions without chloride ions.

**Figure 11.**
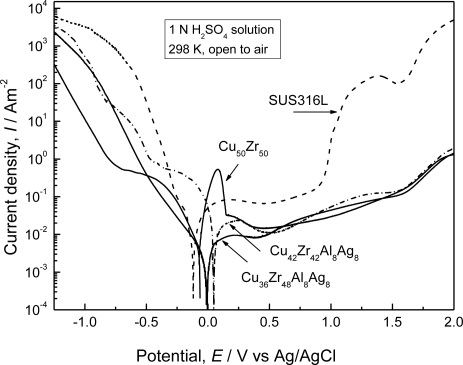
Potentiodynamic polarization curves of the as-cast Cu–Zr–Ag–Al glassy alloys measured in 1 N H_2_SO_4_ solution open to air at 298 K. Polarization curves of the Cu_50_Zr_50_ glassy alloy and a SUS316L stainless steel are also shown for comparison.

Figure 11 shows the potentiodynamic polarization curves of the as-cast Cu*–*Zr*–*Ag*–*Al BMG rods with a diameter of 2 mm in 1 N H_2_SO_4_ solution open to air at 298 K. The polarization curves of the melt-spun Cu_50_Zr_50_ ribbon and a SUS316L stainless steel are also shown for comparison. The binary Cu_50_Zr_50_ glassy alloy shows the active-passive transition and its active dissolution current peak is quite high. For Cu_42_Zr_42_Ag_8_Al_8_ alloy, the active dissolution current peak decreases remarkably with alloying Ag and Al elements to the binary Cu*–*Zr alloy. In addition, spontaneous passivation with a significantly low current density of the order of 10^−2^ A·m^−2^ takes place for Cu_36_Zr_48_Ag_8_Al_8_ alloy. Its passive current density is about one order of magnitude lower than that of the SUS316L. SEM observation showed that no pitting corrosion was observed for (CuZr)-based alloys during anodic polarization. Therefore, the newly developed Cu*–*Zr*–*Ag*–*Al BMGs exhibit high corrosion resistance in 1 N H_2_SO_4_ solution and their corrosion resistance is much better than that of SUS316L.

##### 2.3.1.2. Chemical Characteristics of the Passive Surface Film

**Figure 12.**
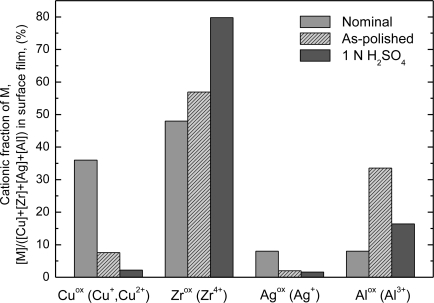
The change in cationic fraction in the surface film for the as-cast Cu_36_Zr_48_Ag_8_Al_8_ alloy exposed to air and that immersed in 1 N H_2_SO_4_ solution open to air for 168 h after mechanical polishing.

In order to clarify the surface-related chemical characteristics of the alloy, XPS analyses were performed for the Cu_36_Zr_48_Ag_8_Al_8_ specimens as-polished mechanically in cyclohexane or immersed for 168 h in 1 N H_2_SO_4_ solution. The valence of oxidized states in the surface film can be identified as Cu^+^, Cu^2+^, Zr^4+^, Ag^+^, and Al^3+^. Figure 12 shows the cationic fraction of elements in the surface film for the Cu_36_Zr_48_Ag_8_Al_8_ alloy exposed to air and a sample immersed in 1 N H_2_SO_4_ solution open to air for 168 h after mechanical polishing [27]. When the alloy is exposed to air after mechanical polishing, Zr and Al cations are enriched in the surface film, whereas Cu and Ag cations are deficient. This is caused by the preferential oxidation of Zr and Al elements during air exposure. When the as-polished alloy is immersed in 1 N H_2_SO_4_ solution for 168 h, a new surface film is formed, which is different from the as-polished film. This is a result of the selective dissolution of Al and Cu elements from the alloy surface film. After immersion, it is found that Zr cationic ions further concentrate at the surface, while Cu and Al cations inversely decrease. Moreover, a small amount of Ag cations are observed in the passive film on the alloy. These results indicate that open circuit immersion for the glassy alloy in 1 N H_2_SO_4_ leads to rapid initial dissolution of Al and Cu with a consequent enrichment in Zr content in the surface film. The thickness of the as-polished surface film and passive surface film is ∼2.8 nm and 3.2 nm, respectively. Consequently, the origin of high corrosion resistance for the present alloy in H_2_SO_4_ solution is explained by formation of the highly protective Zr- and Al-enriched thin surface film, which is able to separate the bulk of the alloy from the corrosive solutions.

Similar XPS results have been found for the Cu–Zr–Ag–Al alloy immersed in 1 N NaOH solution and will not be presented here in detail. In conclusion, the present alloy exhibits high corrosion resistance in acidic and alkaline solutions. However, the main disadvantage of the present alloy is that the alloy shows low corrosion resistance in chloride-ion-containing solution. The surface film of the alloy immersed in chloride-ion-containing solution is less protective, giving rise to accumulation of uneven non-protective corrosion products on the alloy surface. In addition, a larger amount of Cu, Zr, Ag, and Al cations are detected in this solution during the open circuit immersion by ICP-OES measurement [27], which is in agreement with the corrosion rate. Therefore, it is necessary to improve the corrosion resistance of the present alloy system against chloride ions by alloying the corrosion resistant elements or modification of its surface.

#### 2.3.2. The Enhanced Corrosion Resistance of Cu–Zr–Ag–Al–Nb BMGs

**Figure 13.**
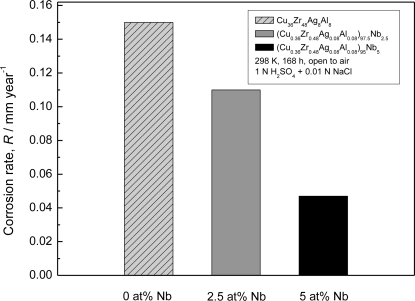
Average corrosion rates of the as-cast (Cu_0.36_Zr_0.48_Ag_0.08_Al_0.08_)_100−x_Nb_x_ (x = 0–5 at.%) alloys in 1 N H_2_SO_4_ + 0.01 N NaCl solution at 298 K open to air.

The as-cast (Cu_0.36_Zr_0.48_Ag_0.08_Al_0.08_)_100−x_Nb_x_ (x = 0–5 at.%) rods with a diameter of 2 mm consisted of a single glassy phase which was evident from a main halo peak without crystalline peaks in their X-ray diffraction patterns. We measured average corrosion rates of the as-cast Cu*–*Zr*–*Ag*–*Al*–*Nb alloy rods immersed in acidic and alkaline solutions open to air at 298 K [28]. No weight loss was detected for all the rod samples after immersion in 1 N H_2_SO_4_ and 1 N NaOH solutions, even after two months. This means that the corrosion rate of the alloy is less than 1 × 10^−3^ mm·y^−1^. After immersion in 1 N H_2_SO_4_ + 0.01 N NaCl solution for one week, as shown in Figure 13, the 0 at.% Nb and 2.5 at.% Nb alloys show the high corrosion rates of 0.15 and 0.11 mm·y^−1^, respectively. In addition, the corrosion resistance of the alloy containing 5 at.% Nb is much improved, and the 5 at.% Nb alloy exhibits a much lower corrosion rate of 0.047 mm·y^−1^. These results reveal that the alloys without Nb and with a small amount of Nb are sensitive to chloride ions. However, when the Nb content is raised to 5 at%, the corrosion resistance against localized corrosion in chloride-ion-containing solutions is greatly enhanced. Apparently, the presence of Nb element in the alloy inhibits the dissolution of the constituent elements into the solutions. Other groups’ work [29,30] also investigated the effect of microalloying of Nb and Ti on corrosion resistance of Cu–Zr–Al–Ag BMGs. Corrosion resistance of CuZr-based BMGs is improved by alloying with Nb or Ti elements in various aqueous solutions.

The nature of passive surface films on the alloys plays a vital role in the mechanism of corrosion resistance. XPS measurements revealed the addition of Nb is favorable for the alloys in forming Zr- and Nb-enriched highly protective surface film with higher chemical stability in 1 N H_2_SO_4_ solution. As compared to the passive surface formed for the Cu_36_Zr_48_Ag_8_Al_8_ alloy after immersion in H_2_SO_4_ solution [27], the passive surface for the 5 at.% Nb alloy is improved by the enrichment of Nb in the surface.

## 3. Conclusions

The corrosion resistance of metallic glasses is superior to that of their crystalline counterparts due to the lack of grain boundaries and second phase precipitates. However, the alloy composition is also a very important factor dominating the corrosion resistance in all materials, including metallic glasses. Alloying additional elements, such as Mo, Nb, Ta and Ni, is effective in improving the corrosion resistance of Cu-based BMGs in various solutions. These alloying elements play different roles in glass-forming ability (GFA) and corrosion resistance for Cu-based BMGs. A better understanding of the role of the alloy constituent elements in corrosion resistance is useful for designing a high corrosion resistant BMG alloy.

## Figures and Tables

**Table 1.** Average corrosion rates of Cu–Hf–Ti–(Mo, Nb, Ta) in chloride containing solutions estimated from weight loss after immersion for 168 h at 298 K.

**Alloys**	**Corrosion rate (mm/year)**		
	**1 N HCl**	**3 mass% NaCl**	**1 N H_2_SO_4_ + 0.001 N NaCl**
Cu_60_Hf_25_Ti_15_	0.340	0.100	0.011
(Cu_06_Hf_0.25_Ti_0.15_)_98_Mo_2_	0.174	4.2 × 10^−3^	<1 × 10^−3^
(Cu_06_Hf_0.25_Ti_0.15_)_98_Nb_2_	0.165	3.2 × 10^−3^	<1 × 10^−3^
(Cu_06_Hf_0.25_Ti_0.15_)_98_Ta_2_	0.166	3.3 × 10^−3^	<1 × 10^−3^
